# Tris(dibenzylideneacetone)dipalladium(0) (Tris DBA) Abrogates Tumor Progression in Hepatocellular Carcinoma and Multiple Myeloma Preclinical Models by Regulating the STAT3 Signaling Pathway

**DOI:** 10.3390/cancers13215479

**Published:** 2021-10-31

**Authors:** Loukik Arora, Chakrabhavi Dhananjaya Mohan, Min Hee Yang, Shobith Rangappa, Amudha Deivasigamani, Alan Prem Kumar, Ajaikumar B. Kunnumakkara, Manoj Garg, Arunachalam Chinnathambi, Sulaiman Ali Alharbi, Tahani Awad Alahmadi, Kanchugarakoppal S. Rangappa, Kam Man Hui, Gautam Sethi, Kwang Seok Ahn

**Affiliations:** 1 Department of Pharmacology, Yong Loo Lin School of Medicine, National University of Singapore, Singapore 117600, Singapore; arora.loukik@u.nus.edu (L.A.); csiapk@nus.edu.sg (A.P.K.); 2 Department of Studies in Molecular Biology, University of Mysore, Manasagangotri, Mysore 570006, India; mohan@biochemistry.uni-mysore.ac.in; 3 KHU-KIST Department of Converging Science and Technology and Department of Science in Korean Medicine, College of Korean Medicine, Kyung Hee University, 24 Kyungheedae-ro, Dongdaemun-gu, Seoul 02447, Korea; didmini@naver.com; 4 Adichunchanagiri Institute for Molecular Medicine, Adichunchanagiri University, BG Nagara, Nagamangala Taluk 571448, India; shobithrangappa@gmail.com; 5 National Cancer Centre, Division of Cellular and Molecular Research, Humphrey Oei Institute of Cancer Research, Singapore 169610, Singapore; amudha.deivasigamani@nccs.com.sg; 6 Cancer Science Institute of Singapore, and Centre for Cancer Research, Yong Loo Lin School of Medicine, National University of Singapore, Singapore 117599, Singapore; 7 Department of Biosciences and Bioengineering, Indian Institute of Technology Guwahati, Guwahati 781039, India; kunnumakkara@iitg.ac.in; 8 Amity Institute of Molecular Medicine and Stem Cell Research (AIMMSCR), Amity University, Noida 201313, India; mgarg@amity.edu; 9 Department of Botany and Microbiology, College of Science, King Saud University, Riyadh 11451, Saudi Arabia; carunachalam@ksu.edu.sa (A.C.); sharbi@ksu.edu.sa (S.A.A.); 10 Department of Pediatrics, College of Medicine, King Saud University, King Khalid University Hospital, P.O. Box 2925, Riyadh 11461, Saudi Arabia; talahmadi@ksu.edu.sa; 11 Institution of Excellence, Vijnana Bhavan, University of Mysore, Manasagangotri, Mysore 570006, India; rangappaks@gmail.com

**Keywords:** Tris DBA, STAT3 signaling inhibitor, orthotopic, xenograft, SHP2

## Abstract

**Simple Summary:**

STAT3 is a major oncogenic transcription factor that is constitutively activated in many types of human cancers, including hepatocellular carcinoma (HCC) and multiple myeloma (MM). Many STAT3 inhibitors have gained momentum in clinical trials towards the treatment of various cancers. In the present study, we have investigated the STAT3 inhibitory efficacy of Tris DBA, a palladium-based compound, in HCC and MM cancer cells and preclinical cancer models. Tris(dibenzylideneacetone)dipalladium(0) (Tris DBA) abrogated the STAT3 signaling pathway in both models by elevating the expression of SHP2. Functionally, Tris DBA inhibited cell proliferation, migration, invasion, and regressed tumor metastasis. Although many studies propose Tris DBA as a modulator of MAPK, Akt, phospho-S6 kinase, and N-myristoyltransferase-1, we have comprehensively demonstrated for the first time that Tris DBA is an inhibitor of STAT3 signaling in preclinical cancer models. These results support the consideration of Tris DBA in clinical trials in translational relevance.

**Abstract:**

STAT3 is an oncogenic transcription factor that controls the expression of genes associated with oncogenesis and malignant progression. Persistent activation of STAT3 is observed in human malignancies, including hepatocellular carcinoma (HCC) and multiple myeloma (MM). Here, we have investigated the action of Tris(dibenzylideneacetone) dipalladium 0 (Tris DBA) on STAT3 signaling in HCC and MM cells. Tris DBA decreased cell viability, increased apoptosis, and inhibited IL-6 induced/constitutive activation of STAT3, JAK1, JAK2, and Src in HCC and MM cells. Tris DBA downmodulated the nuclear translocation of STAT3 and reduced its DNA binding ability. It upregulated the expression of SHP2 (protein and mRNA) to induce STAT3 dephosphorylation, and the inhibition of SHP2 reversed this effect. Tris DBA downregulated the expression of STAT3-driven genes, suppressed cell migration/invasion. Tris DBA significantly inhibited tumor growth in xenograft MM and orthotopic HCC preclinical mice models with a reduction in the expression of various prosurvival biomarkers in MM tumor tissues without displaying significant toxicity. Overall, Tris DBA functions as a good inhibitor of STAT3 signaling in preclinical HCC and MM models.

## 1. Introduction

Cancer is a prominent public health concern and the second major cause of death globally after cardiovascular disease and both its incidence and mortality rates are increasing every year [1,2,3]. Hepatocellular carcinoma (HCC) is the leading type of liver cancer, affecting 0.8 million people in 2012 throughout the globe [4], and the five-year survival rate has been estimated to be between 5% and 14%, which is critically low [5]. Hepatitis B infection remains the leading risk factor of HCC followed by non-alcoholic fatty liver disease, alcoholic hepatitis, aflatoxin B1 intake, and hemochromatosis [6,7]. HCC may be often detected at the metastatic stage, which drastically reduces the treatment efficacy and options [8,9]. Multiple myeloma (MM) stands second among hematological malignancies in Western countries, contributing approximately 10% of hematological malignancies [10,11,12]. Although precise risk factors are not clearly listed, factors such as age, gender, race, and family history are often believed to contribute to the development of MM [13].

Signal transducer and activator of transcription 3 (STAT3) can be aberrantly activated in solid and hematological cancers including HCC and MM respectively [14,15,16,17]. Several studies have demonstrated the critical role of STAT3 in malignant transformation and progression. The STAT family comprises seven members, namely STAT1, STAT2, STAT3, STAT4, STAT5a, STAT5b, and STAT6 [18,19,20,21]. STAT1, STAT3, and STAT5 are widely studied in the cancer context, among which STAT3 stands in the forefront [22,23,24]. STAT3 is predominantly activated by IL-6 family cytokines transiently in non-diseased conditions. In canonical signaling, the binding of IL-6 to its receptor results in the activation of gp130 and other nonreceptor tyrosine kinases (NRTKs), such as JAK and Src [25]. The phosphorylated NRTKs or some of the activated receptor tyrosine kinases (such as EGFR) phosphorylates STAT3^Y705^ and activates it [26,27,28]. The phosphorylated monomer undergoes dimerization with another monomer to translocate into the nucleus through specific importin [29,30]. STAT3-binding sites have been identified in the promoter region of several genes that are involved in cell growth and proliferation, inflammation, prosurvival, antiapoptosis, proangiogenic, and metastasis [31,32,33]. The overexpression of STAT3 targeted genes due to its persistent activation in cancers provides enormous growth potential to the cancer cells and encourages the advancement of the disease. Therefore, mitigation of the STAT3 cascade may provide a significant clinical benefit to patients. On the other hand, the phosphorylation state of proteins of STAT3 signaling is modulated by protein tyrosine phosphatases (PTPs), such as SHP1, SHP2, TC-PTP, PTP1B, CD45, and PTPRT. Among these proteins, SHP2 is one of the major ubiquitously expressed tyrosine phosphatases with two SH2 domains and a phosphatase domain. SHP2 interacts with phosphorylated proteins of the STAT3 pathway (such as cytokine receptor complexes, JAKs, and Src family kinases) and dephosphorylate them through its phosphatase activity to abrogate downstream signaling. Studies have indicated that the hepatocyte-specific deletion of SHP2 led to the promotion of STAT3 signaling and thereby hepatic inflammation and tumor progression in mice [34].

Tris DBA is an organometallic complex compound where two palladium atoms are bound to the alkene units of three dibenzylideneacetone [35]. The compound is moderately soluble in organic solvents. The antitumor potential of Tris DBA in a few cancer models has been analyzed previously. Bhandarkar and colleagues demonstrated that Tris DBA can reduce melanoma cell proliferation by inhibiting the activation of MAPK, Akt, STAT3, and phospho-S6 kinase, and downregulated the expression of N-myristoyltransferase-1 [36]. Tris DBA decreased proliferation and showed an additive cytotoxic effect with proteasome inhibitors, such as bortezomib and carfilzomib, towards MM cells [37]. In addition, Tris DBA regulated the expression of the Bcl-2 family proteins by targeting ribosomal protein S6 in primary chronic lymphocytic leukemia B-cells [38]. Tris DBA has been proposed to target N-myristoyltransferase-1 to induce growth-inhibitory and antimetastatic activity in pancreatic cancer cells [39]. These findings have established Tris DBA as a promising anticancer agent, although its precise mechanism of action is yet to be explored. In the present study, we have evaluated the effect of Tris DBA on STAT3 signaling in HCC and MM cells and its antitumor efficacy in preclinical settings.

## 2. Materials and Methods

### 2.1. Reagents

**Figure 1:**
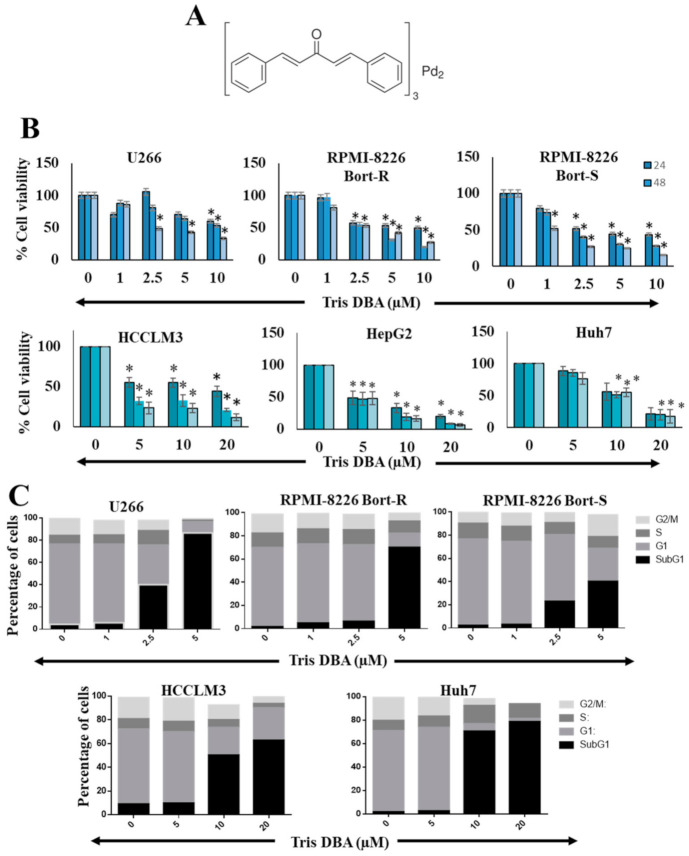
Tris DBA decreases cell viability. (**A**) The structure of Tris DBA. (**B**) The efficacy of Tris DBA on the viability of MM (U266, and RPMI-8226) and HCC (HCCLM3, HepG2, and Huh7) cells was examined by MTT assay at different time-points and concentrations. Bars indicate standard deviation; * *p* < 0.05 versus no treatment (no treatment is 0.1% DMSO). The experiments were repeated thrice. (**C**) MM (U266, and RPMI-8226) and HCC (HCCLM3, HepG2, and Huh7) cells were incubated with Tris DBA and analyzed using flow cytometry to determine the distribution of cells across the cell cycle.

Tris DBA, MTT, Tris, glycine, NaCl, SDS, BSA, IL-6, EGF, and pervanadate were procured from Sigma-Aldrich. The structure of Tris DBA is provided in Figure 1A. The stock solution of Tris DBA (10 mmol/L stock) was prepared in dimethylsulfoxide (DMSO) and stored at 4 °C. Tris DBA was further diluted with a cell culture medium as per the requirements. Cell culture media (Dulbecco’s Modified Eagle Medium (DMEM), and Roswell Park Memorial Institute (RPMI)), and fetal bovine serum (FBS) were purchased from Life Technologies. Rabbit or mouse monoclonal and polyclonal antibodies against Bak, Bcl-2, PARP, survivin, Mcl-1, SHP-2, and PTP1B were obtained from Santa Cruz Biotechnology. Antibodies against phospho-STAT3 (Tyr705), STAT3, phospho-JAK1/2, JAK1/2, caspase-3, phospho-Src, Src, cyclin D1, SHP1, and β-Actin were purchased from Cell Signaling Technology. The BioRender program was used to generate the graphical abstract of this paper.

### 2.2. Cell Lines

HepG2 cell line was obtained from American Type Culture Collection. Huh-7 cell line was obtained from the Japanese Collection of Research Bioresources (JCRB) Cell Bank (Osaka, Japan), and the HCCLM3 cell line was a kind gift from Professor Zhao-You Tang at the Liver Cancer Institute (Zhongshan Hospital, Fudan University, Shanghai, China). MM cells (U266 and RPMI-8226 (both bortezomib resistant and sensitive)) were a kind gift from Prof. Wee Joo Chng (Cancer Science Institute, Singapore). HCC cells were cultured in Dulbecco’s modified Eagle medium (DMEM) with 10% FBS. All MM cell lines were cultured in RPMI-1640 medium with 10% FBS.

### 2.3. MTT Assay

The cytotoxic effect of Tris DBA towards MM and HCC cells was evaluated by the in vitro cytotoxicity assay using MTT dye as reported earlier [40,41]. In brief, HCC and MM cells were treated with indicated concentrations of Tris DBA in a 96-well plate for different time intervals up to 72 h at 37 °C. MTT solution (20 μL, 5 mg/mL in PBS) was added to each well and incubated for 2 h at 37 °C, followed by the addition of 0.1 mL of DMSO. This mixture was incubated overnight at 37 °C and the optical density of the resultant product was measured at 570 nm.

### 2.4. Annexin V Assay

Flow cytometric analysis was performed to determine the extent of apoptosis in MM and HCC cells using Annexin V and PI staining as described previously [42]. HCC and MM cells were propagated overnight in a cell culture vessel followed by treatment with Tris DBA at indicated doses for the given time points. These cells were harvested and suspended for 15 min in a solution containing FITC-tagged annexin V and PI. The stained cells were immediately processed and examined using a CyAn ADP flow cytometer.

### 2.5. Cell Cycle Analysis Using Flow Cytometry 

The alignment of tumor cells in different phases of the cell cycle was analyzed using flow cytometry by staining with PI as reported earlier [43,44]. To examine the effect of Tris DBA on the cell cycle distribution, the cancer cells were treated with indicated doses of Tris DBA for a given duration, and cells were harvested. The cell pellet was suspended in propidium iodide (2.5 µg/mL) and RNAse (0.5 mg/mL RNase A in PBS) after treatment and analyzed using a CyAn ADP flow cytometer (Dako Cytomation).

### 2.6. Western Blotting

The cells were incubated with Tris DBA for given time points at given doses and lysed used for western blotting experiments as described previously [45]. Briefly, Tris DBA-treated whole-cell extracts were prepared using lysis buffer (20 mM Tris (pH 7.4), 250 mM NaCl, 2 mM EDTA (pH 8.0), 0.1% Triton X-100, 0.01 mg/mL aprotinin, 0.005 mg/mL leupeptin, 0.4 mM PMSF, and 4 mM NaVO_4_). Lysates were centrifuged and the supernatant was resolved on SDS-PAGE. The proteins were transferred into the nitrocellulose membrane and treated overnight at 4 °C with primary antibodies raised against the protein of interest. Thereafter, the nitrocellulose membrane was washed and exposed to secondary antibodies followed by examination using chemiluminescence (ECL; GE Healthcare, Chicago, IL, USA). The uncropped western blot figures can be found in Figure S1.

### 2.7. DNA Binding Assay

The effect of Tris DBA on the DNA interaction ability of STAT3 was assessed using the TransAM assay kit (Active Motif, Carlsbad, CA, USA). The experiment was done as per the directions from the manufacturer as described previously [33,46]. Briefly, nuclear extracts (5 µg) from Tris DBA treated cells were incubated in a 96-well plate coated with an oligonucleotide containing the STAT3-specific DNA probe. Bound STAT3 was then detected by a specific primary antibody. An HRP-conjugated secondary antibody was subsequently applied to detect the bound primary antibody and provided the basis for colorimetric quantification. The enzymatic product was measured at 450 nm with a microplate reader (Tecan Systems, San Jose, CA, USA). The specificity of this assay was tested by the addition of wild-type or mutated STAT3 consensus oligonucleotide in the competitive or mutated competitive control wells before the addition of the nuclear extracts.

### 2.8. Immunocytochemistry for STAT3 Distribution

The cells were seeded in Ibidi glass-bottom dishes in complete medium and treated with Tris DBA or vehicle control for 2 h, then fixed with 10% buffered formalin after washing with PBS. These cells were treated with Triton-X 100 for permeabilization followed by blocking with goat serum (5%) for 1 h. Thereafter, the preparation is treated with monoclonal mouse anti-STAT3 antibody (1:100 dilution) overnight at 4 °C followed by rinsing with PBS and incubated in goat anti-mouse secondary antibody (Alexa-Fluor 568) for 1 h. The cells were then washed and counterstained with DAPI to stain the nuclei. The cells were analyzed immediately under a confocal microscope (Nikon A1).

### 2.9. RNA Isolation and Reverse Transcription-Polymerase Chain Reaction (RT-PCR)

The cells were incubated with Tris DBA, washed, and suspended in Trizol reagent (Invitrogen, Carlsbad, CA, USA) and reverse transcribed as discussed earlier [47]. Initially, total cellular RNA from untreated and Tris DBA-treated cells was isolated using TRIzol reagent using a standard protocol. Thereafter, 1 μg of extracted RNA was used for reverse transcription to generate cDNA of target gene products. For this, 1 μg of RNA sample was reverse transcribed with 1.1 U/mL MultiScribe reverse transcriptase in the presence of suitable buffer, dNTPs, RNase inhibitor, and RNase-free sterile water.

### 2.10. Real-Time Quantitative PCR

Total RNA was isolated from the cells using Trizol reagent and subjected to real-time quantitative PCR analysis as reported previously [48]. For real-time PCR, 100 ng/μL of total RNA was transcribed. For a 50 μL reaction, 10 μL of RT product was mixed with 1X TaqMan Universal PCR Master Mix, 2.5 μL of 20× TaqMan probes for SHP2. GAPDH TaqMan probe was used as input control for each gene of interest. Real-time PCR was programmed as follows: 50 °C for 2 min, 95 °C for 10 min, followed by 40 cycles of denaturation at 95 °C for 15 s and extension at 60 °C for 1 min. The output is interpreted using Sequence Detection Software version 1.3 obtained from Applied Biosystems. Normalization was performed using endogenous GAPDH before relative gene expression studies. Analysis of the difference in threshold cycle (Ct) between treated and untreated cells was performed.

### 2.11. Transfection with SHP2 siRNA, STAT3 siRNA, and STAT3C

We used the Neon™ Transfection System (Invitrogen, Carlsbad, CA, USA) to transfect cells with SHP2 siRNA, STAT3 siRNA, and STAT3C. Briefly, cells and SHP2 siRNA (100 ng/μL), STAT3 siRNA (100 nM), or STAT3C (300 ng) were taken into a clean, dry, sterile centrifuge tube and the following aspiration of cell-siRNA cocktail using Neon Tip in Neon Pipette to restrain the formation of air bubbles. Thereafter, the Neon Pipette was placed into the Neon Tube containing Neon Electrolytic Buffer E. Next cells were subjected to a pulse of 1150 voltage with a width of 30 for transfection. Then, cells were incubated with Tris DBA 48 h post-transfection, and the cell lysate was prepared and analyzed for various proteins.

### 2.12. In Vitro Migration Assay

This experiment was performed to determine the action of Tris DBA on migration as described before [49,50]. For this, HCC cells were propagated in a 6-well plate and a small scratch was made using a pipette tip. The cells were incubated with the indicated dose of Tris DBA for 8 h before treating with CXCL12 (100 ng/mL). The distance between the cell lanes was measured in the control and Tris DBA-treated in the presence and absence of CXCL12. Graphs were plotted against the percentage of migration distance the cells moved before and after treatment, normalized to control.

### 2.13. Invasion Assay

This assay was carried out to assess the anti-invasive property of Tris DBA on HCC and MM cells using the Bio-Coat Matrigel invasion assay system (BD Biosciences, San Jose, CA, USA) as elaborated upon previously [51]. HCC cells were suspended in serum-free DMEM and seeded into the invasion chamber. Thereafter, these cells were incubated with a given dose of Tris DBA for 8 h. In another set of experiments, HCC cells were induced with CXCL12 in the presence and absence of indicated concentrations of Tris DBA. Following incubation, the peripheral surface of the transwell chamber was carefully wiped with cotton swabs, fixed, and subjected to crystal violet staining. The invading cells were then quantified in five randomly selected areas under microscopic observation.

### 2.14. In Vivo Acute Toxicity Studies 

All the in vivo acute toxicity experiments were carried out as per the approved procedures by the SingHealth Institutional Animal Use and Care Committee (protocol number: 2013/SHS/870). Eight-week-old NCr nude male mice (Invivos, Singapore) were used to perform toxicity-related experiments. The experimental animals were administered intraperitoneally with 0.1% DMSO or 25, 50, 100, and 200 mg of Tris DBA. The experimental animals were monitored regularly for any changes in feed and water consumption, body weight, behavior, physical appearance, movement, and other criteria up to the eighth day. On the last day, animals were sacrificed by cardiac puncture, and blood was collected for further analysis for biochemical analysis.

### 2.15. Xenograft MM Mouse Model

In vivo xenograft tumor experiments were carried out as per the approved procedures by KHU Institutional Animal Care and Use Committee (KHUASP(SE)-17-110). The subcutaneous xenograft MM mouse model was established by injecting U266 cells to the right flank of six-week-old athymic *nu/nu* female mice (NARA Biotech, Seoul, Korea). The mice were randomly distributed into three groups (*n* = 6/group) upon tumors reaching the size of 0.25 cm. The first group served as control which was intraperitoneally administered with PBS (200 μL, i.p. thrice/week). The second and third groups intraperitoneally received Tris DBA (50 mg/kg, thrice/week and 100 mg/kg, thrice/week, respectively) for four weeks. All mice were sacrificed after one week and primary tumors were collected for subsequent analysis.

### 2.16. Immunohistochemical Analysis of MM Tumor Samples

The tumors collected from xenograft experiments were processed using phosphate-buffered formalin (10%) and impregnated in the paraffin blocks. Thereafter, they were subjected to cutting, deparaffinization using xylene, and dehydration using graded alcohol. The slides were boiled in sodium citrate (10 mM, pH 6.0) for 30 min to retrieve antigens. This is followed by immunohistochemistry using the kit obtained from Vector Laboratories (ImmPRESS^TM^ Reagent Kit). In brief, hydrogen peroxide (3%) and blocking reagent were used to quench tissue-derived peroxidases and to block non-specific interactions. Further, sections were treated with various antibodies overnight. The next day, slides were repeatedly rinsed with PBS and incubated with ImmPRESS^TM^ reagent. To identify immunoreactive species, 3, 3-diaminobenzidine tetrahydrochloride (DAB) reagent was used as a substrate. Next, Gill’s hematoxylin was used to counterstain the sections followed by the photography of sections using the Olympus microscope (20×). Positive cells appeared brown and were quantified using the Image-Pro Plus 6.0 software package (Media Cybernetics, Inc. Rockville, Maryland)

### 2.17. Orthotopic HCC Tumor Model

In vivo orthotopic tumor experiments were carried out as per the approved procedures by the SingHealth Institutional Animal Use and Care Committee (protocol number: 2013/SHS/870) and as reported earlier [52,53]. The orthotopic HCC mouse model was established by placing a small piece of tumor-derived from HCCLM3-Luc cells. The mice were randomly distributed into three groups upon the bioluminescence signal reaching 10^6^. The first group served as control which was intraperitoneally administered with 0.1% DMSO (thrice/week). The second and third groups intraperitoneally received Tris DBA (50 mg/kg, thrice/week and 200 mg/kg, thrice/week, respectively) for four weeks. The tumor progression/regression was recorded weekly twice by quantifying the bioluminescence signals. Experimental animals were sacrificed by CO_2_ inhalation. Tumor tissues were harvested and stored at −80 °C for further processes.

### 2.18. Statistical Analysis

The obtained numerical data are presented as the mean ± SD unless otherwise mentioned. Student’s *t*-test or one-way ANOVA were employed to measure statistical significance.

## 3. Results

### 3.1. Tris DBA Decreased the Viability and Increased the subG1 Population of HCC and MM Cells

We deciphered the action of Tris DBA on the viability of HCC and MM cells by MTT assay. For this, we used HCC (HCCLM3, Huh7, HepG2), MM (U266), and bortezomib-resistant/sensitive MM (RPMI-8226) cell lines. Tris DBA significantly decreased the viability of all the tested cells, thus indicating the cytotoxic effect of Tris DBA on both types of cancer cells (Figure 1B). Flow cytometric analysis revealed that the treatment with Tris DBA significantly increased the subG1 cell population up to 80% in HCC and MM cells, indicating that the cell cycle progression is disrupted (Figure 1C). However, the subG1 cell population is relatively less (30%) in bortezomib-resistant RPMI-8226 cells.

### 3.2. Tris DBA Induced Apoptosis of HCC and MM Cells 

**Figure 2:**
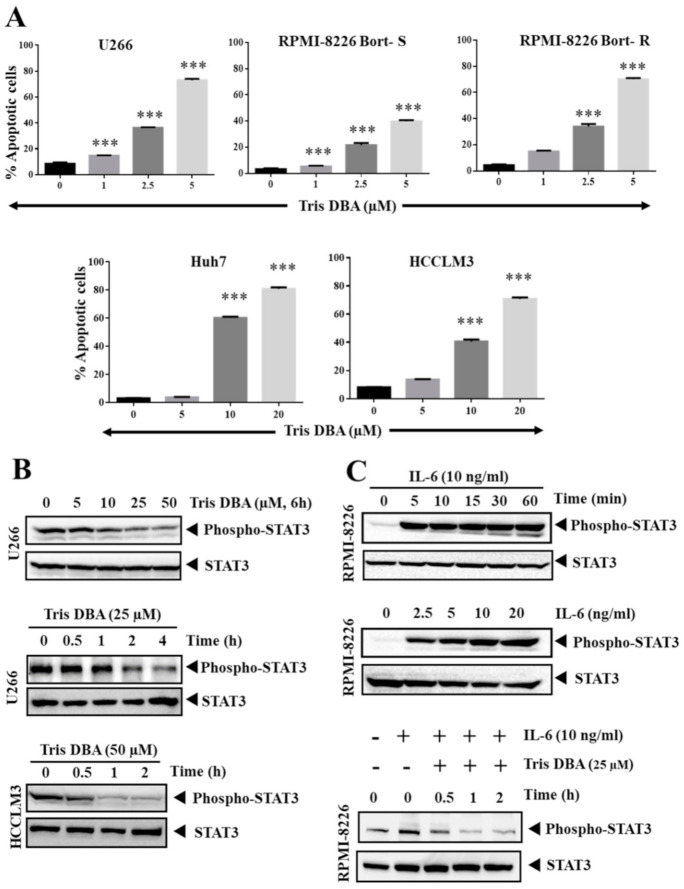
Tris DBA induces apoptosis and mitigates the STAT3 activation. (**A**) MM (U266, and RPMI-8226) and HCC (Huh7 and HCCLM3) cells were treated with Tris DBA and treated with FITC-Annexin V and PI to quantify the early and late apoptotic cells. Bars indicate standard deviation; *n* = 3; *** *p* < 0.001 versus no treatment. (**B**) U266 and HCCLM3 cells were exposed to Tris DBA at indicated concentrations and time points and cell lysates were used for SDS-PAGE and Western blotting to analyze phospho-STAT3/STAT3 expression. (**C**) RPMI-8226 cells were incubated with Tris DBA (25 µM) at given time points and induced with IL-6 (10 ng/mL). The cell lysates were used for SDS-PAGE and Western blotting to analyze phospho-STAT3/STAT3 expression.

We performed FITC-Annexin V-PI staining to verify that the action of Tris DBA is inducing apoptosis. We observed a dose-dependent elevation in the percentage FITC-Annexin V-PI-positive cells. This result indicated that the number of apoptotic cells significantly increases in all the tested cell lines (Figure 2A). Tris DBA also significantly increased the cell population in both bortezomib-resistant and bortezomib-sensitive MM cells.

### 3.3. Tris DBA Altered STAT3 Phosphorylation in Tumor Cells

Initially, we examined the effect of Tris DBA on the activation of STAT3 in HCC and MM cells. Tris DBA significantly reduced the constitutively activated STAT3 in U266 and HCCLM3 cells (Figure 2B). RPMI-8226 has a basal level of phospho-STAT3^Y705^ expression and treatment of these cells with IL-6 triggered the phosphorylation of STAT3^Y705^ in a time- and dosage-dependent manner (Figure 2C, upper panels). IL-6 is a potent stimulator of STAT3 signaling in the tumor microenvironment. The treatment with Tris DBA resulted in the suppression of IL-6 driven STAT3 activation without a change in the total STAT3 expression (Figure 2C, lower panel). 

### 3.4. Tris DBA Decreased Nuclear Localization and Ablated the Binding of STAT3

**Figure 3:**
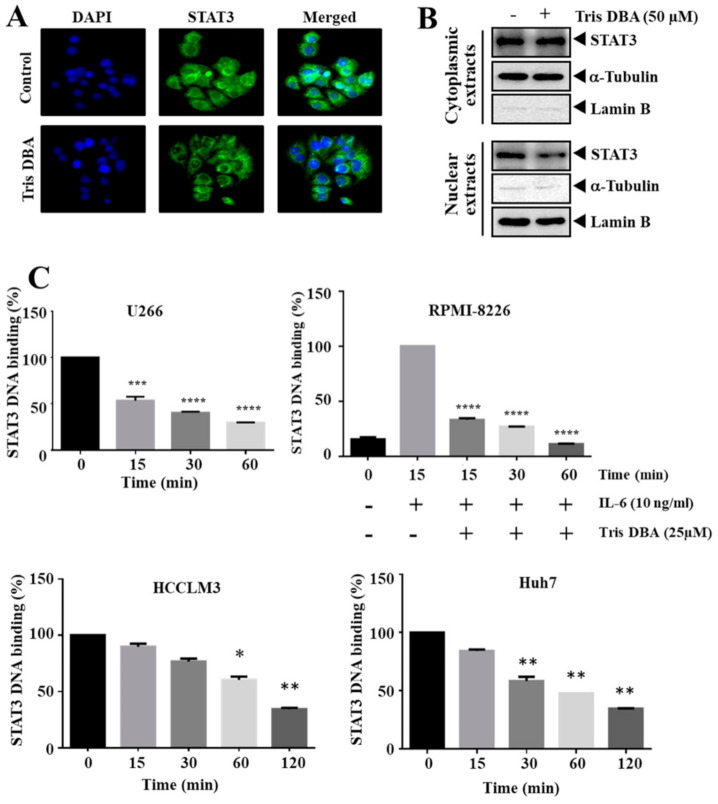
Tris DBA downregulates nuclear STAT3 and its DNA binding ability. (**A**) HCCLM3 cells were treated with Tris DBA (50 µM) and examined for the cellular compartmentalization of STAT3 by immunocytochemistry. DAPI was used to counterstain and visualize nuclei. (**B**) Cells were treated as described above. Cytoplasmic extracts and nuclear extracts were subjected to western blotting. α-tubulin was used as a loading control for the cytoplasmic extracts and lamin B was used as a loading control for the nuclear extracts. (**C**) MM (U266, IL-6 induced RPMI-8226), and HCC (HCCLM3, and Huh7) cells were incubated with Tris DBA (25 and 50 µM respectively) and nuclear extracts were prepared to analyze DNA interaction of STAT3 as described in methods. Bars indicate standard deviation; *n* = 3; * *p* < 0.05, ** *p* < 0.01, *** *p* < 0.001, **** *p* < 0.0001 versus no treatment.

Next, we deciphered the action of Tris DBA on the cellular distribution of STAT3 in HCCLM3 cells. Tris DBA downregulated the nuclear pool of STAT3 in HCCLM3 cells compared with vehicle-treated samples as demonstrated by the results of immunocytochemistry (Figure 3A). Nuclear translocation is essential to execute the transcription of target genes. Further, we prepared the nuclear extract of DMSO/Tris DBA treated cells (Figure 3B) and analyzed whether Tris DBA also influences the DNA interaction ability of STAT3. We observed a significant reduction in the DNA interaction ability of STAT3 in different tumor cells (Figure 3B). We also observed inhibition of IL-6 triggered DNA binding ability of STAT3 in RPMI-8226 cells (Figure 3C), thus indicating that Tris DBA may interfere with STAT3 activation and subsequent gene transcription.

### 3.5. Tris DBA Inhibited the Phosphorylation of Upstream Kinases of STAT3 Signaling

**Figure 4:**
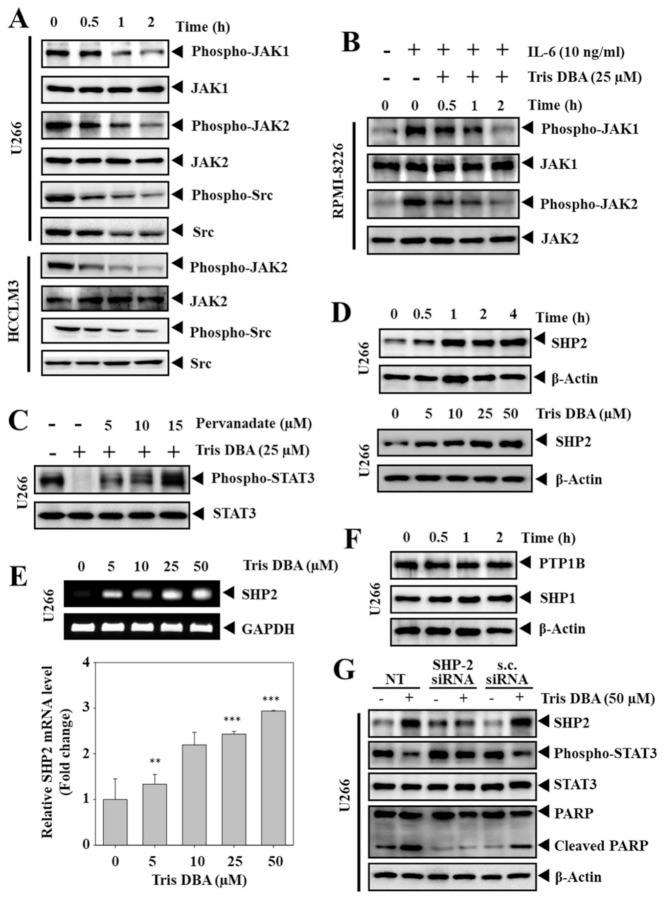
Tris DBA suppresses upstream proteins and enhances the expression of SHP2. (**A**) U266 and HCCLM3 cells were exposed to Tris DBA (25 and 50 µM respectively) and cell lysates were used to analyze phosphorylated NRTKs and total proteins expression. (**B**) RPMI-8226 cells were exposed to Tris DBA (25 µM) at indicated time points and stimulated with IL-6 (10 ng/mL). The cell lysates were used to analyze phosphorylated NRTKs and total protein expression. (**C**) U266 cells were treated with Tris DBA (25 µM) and pervanadate at indicated doses to analyze phosphorylated STAT3 and total STAT3 expression. (**D**,**F**) Tris DBA treated U266 cells were lysed and analyzed for levels of various phosphatases. (**E**) Tris DBA treated U266 cells were lysed and used for the analysis of SHP2 mRNA expression. GAPDH was used as input control. Bars indicate standard deviation; *n* = 3; ** *p* < 0.01, *** *p* < 0.001 versus no treatment. (**G**) U266 cells were transfected with SHP2 or scrambled siRNA (100 nM) for 48 h. After that, cells were treated with Tris DBA (50 μM) and western blotting for SHP2, phospho-STAT3, STAT3, and PARP was carried out.

JAK1, JAK2, Src, and EGFR are the major upstream kinases that are involved in relaying signals for the STAT3 activation. Next, we measured the potential of Tris DBA treatment on the phosphorylation of upstream kinases regulating STAT3 signaling, such as JAK1, JAK2, and Src in HCC and MM cells. We observed a significant decline in the constitutive activation of JAK1, JAK2, Src in U266 cells, and JAK2, Src in HCCLM3 cells (Figure 4A). We also investigated the effect of Tris DBA on IL-6-induced phosphorylation of JAK1 and JAK2 in RPMI-8226 cells. We observed a substantial reduction in the IL-6 induced activation of JAK1 and JAK2 (Figure 4B), thereby indicating that Tris DBA suppresses the activity of upstream kinases to modulate STAT3 functions. 

### 3.6. Tris DBA Altered the Expression of SHP2

We further determined the action of pervanadate on Tris DBA-induced STAT3 inhibition in MM cells. The exposure to pervanadate reversed the Tris DBA-induced STAT3 inhibition, thereby indicating the involvement of PTPs (Figure 4C). The action of Tris DBA on the cellular levels of major PTPs, such as SHP1, SHP2, and PTP1B, was measured. Interestingly, Tris DBA elevated the protein levels of SHP2 in a dosage- and time-dependent fashion (Figure 4D). We also observed that treatment with Tris DBA increased the mRNA expression of SHP2, thus indicating that this protein may be regulated at the transcriptional level (Figure 4E). However, a substantial change was not noted in the expression of SHP1 and PTP1B proteins (Figure 4F).

### 3.7. Depletion of SHP2 Reversed the Tris DBA Induced STAT3 Inhibition

We transfected U266 cells with siRNA direct towards SHP2 to study the specificity of Tris DBA towards the STAT3 signaling pathway. The treatment of Tris DBA alone decreased STAT3 phosphorylation with a corresponding increase in SHP2. The deletion of SHP2 using siRNA resulted in the restoration of STAT3 phosphorylation and Tris DBA did not affect the STAT3 activation in SHP2-depleted cells (Figure 4G). The cells treated with scrambled siRNA served as control. These results substantiated the role of SHP2 in mediating the STAT3 inhibitory actions of Tris DBA. 

### 3.8. Tris DBA Altered the Expression of Oncogenic Proteins 

**Figure 5:**
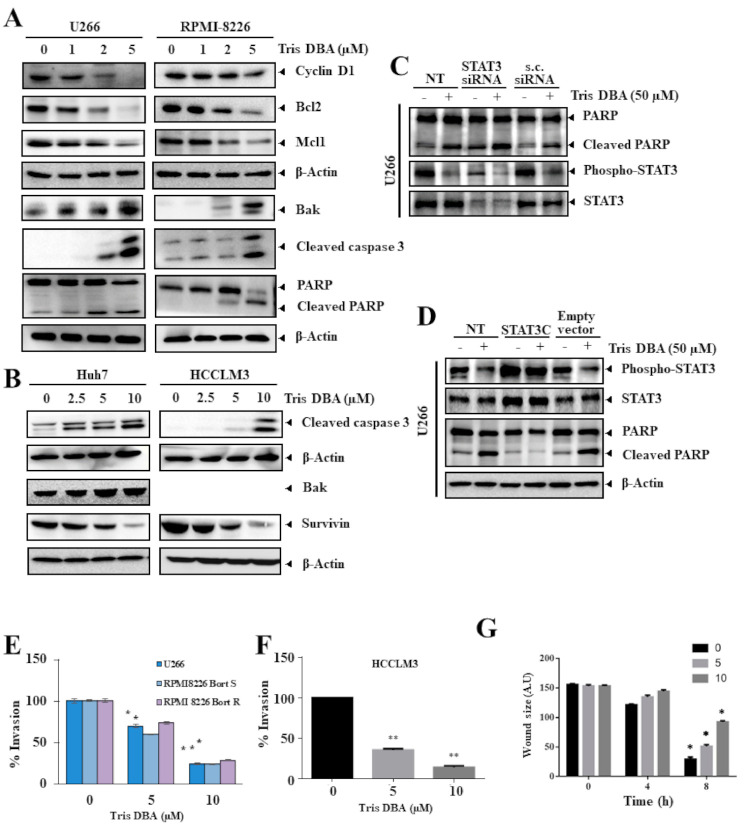
Tris DBA downmodulates STAT3 targeted gene expression and induces apoptosis. (**A**,**B**) MM (U266, and RPMI-8226) and HCC (Huh7, and HCCLM3) cells were incubated with given doses of Tris DBA and cell lysates were analyzed for the expression of cyclin D1, Bcl2, Mcl1, Bak, caspase 3, survivin, and PARP expression. (**C**) U266 cells were transfected with scrambled or STAT3 siRNA (100 nM). After 48 h, cells were treated with Tris DBA 50 μM and the western blotting was done. (**D**) Cells were transfected with STAT3C or empty vector (300 ng). After 48 h, cells were treated with Tris DBA 50 μM and western blot analysis for various proteins was carried out. (**E**) Tris DBA suppresses cancer cell invasion and migration. MM (U226, and RPMI-8226) and HCCLM3 cells were incubated with different doses of Tris DBA and seeded in invasion chambers in serum-free media. The lower chamber was filled with FBS supplemented media. After the incubation with the suggested time point, the cells on the upper side were stained with crystal violet to visualize and quantify them. (**F**) HCCLM3 cells were seeded into a culture vessel that has a culture-insert and the removal of this insert creates the cell-free space. The movement of the cell is visualized and measured either on treatment with Tris DBA or untreated samples. (**G**) HCCLM3 cells were cultured in a culture dish, a cell-free space (wound) is created and cells were treated with Tris DBA. Thereafter, the antimigratory effect of Tris DBA was examined at different time-points. Bars indicate standard deviation; *n* = 3; * *p* < 0.05, ** *p* < 0.01 versus no treatment.

We determined the action of Tris DBA on the expression of STAT3 driven proteins in MM (Figure 5A) and HCC cells (Figure 5B). Consistent with the inhibition of STAT3 phosphorylation, Tris DBA downregulated the expression of oncogenic factors, such as cyclin D1, Bcl-2, Mcl1, and survivin, with an upregulation of proapoptotic factors such as Bak protein in the tested cell lines. Furthermore, Tris DBA also promoted the cleavage of caspase-3 and PARP, thus indicating that Tris DBA treated cells are undergoing apoptosis (Figure 5A,B).

### 3.9. Knock-Down or Overexpression of STAT3 Modulates Tris DBA-Promoted Apoptosis

We further determined whether the inhibition of STAT3 phosphorylation was crucial in Tris DBA-induced apoptosis. U266 cells were transfected with STAT3 siRNA or STAT3C for knock-down or overexpression of STAT3. As shown in Figure 5C, Tris DBA-induced apoptosis was promoted upon transfection with STAT3 siRNA as compared with the scrambled siRNA. However, Tris DBA-induced apoptosis was abolished upon transfection with STAT3C (Figure 5D). These results suggest that the induction of Tris DBA-induced apoptosis is mediated at least in part through the mitigation of the STAT3 signaling pathway.

### 3.10. Tris DBA Significantly Modulated the Migratory and Invasive Potential

STAT3 target gene products have been reported to be entangled with the migration and invasion of cancer cells. Next, we measured the outcome of Tris DBA on cellular motility using invasion and migration assay systems. Tris DBA significantly suppressed the invasion of U266, RPMI-8226 (sensitive and resistant), and HCCLM3 cells in a concentration-dependent fashion (Figure 5E,F). In addition, significant anti-migratory effects were observed in Tris DBA treated HCCLM3 cells (Figure 5G).

### 3.11. Tris DBA Did Not Display Toxicity in Preclinical Studies

**Figure 6:**
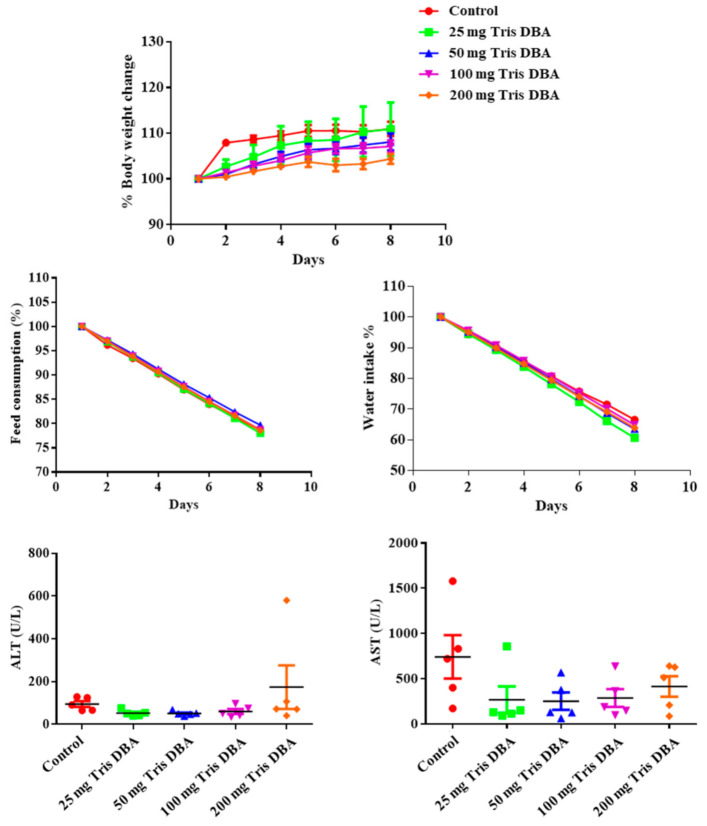
Tris DBA exhibited no significant toxicity in vivo. The nude mice (*n* = 5) per group were treated with one single dose of Tris DBA (25, or 50, or 100, or 200 mg/kg) and 0.1% DMSO control intraperitoneally and effect of Tris DBA on body weight, feed consumption, water intake, and various biochemical parameters were measured.

We next determined the sub-lethal doses of Tris DBA to perform in vivo tumor studies by measuring its toxicity. For this, male NCr nude mice were injected with Tris DBA (25, 50, 100, and 200 mg/kg/day) for eight days and experimental animals were regularly monitored. The mice group which received DMSO (0.1%) served as control. We did not observe significant changes in Tris DBA treated group of animals relative to the DMSO treated group in terms of body weight, water, and feed consumption (Figure 6). Notably, no significant alterations were observed in the activity of liver function marker enzymes, such as alanine aminotransferase (ALT) and aspartate aminotransferase (AST) (Figure 6), thereby suggesting that Tri DBA did not exhibit significant toxicity in tested animals.

### 3.12. Tris DBA Imparted Antitumor Effect in Xenograft MM and Orthotopic HCC Mice Models

**Figure 7:**
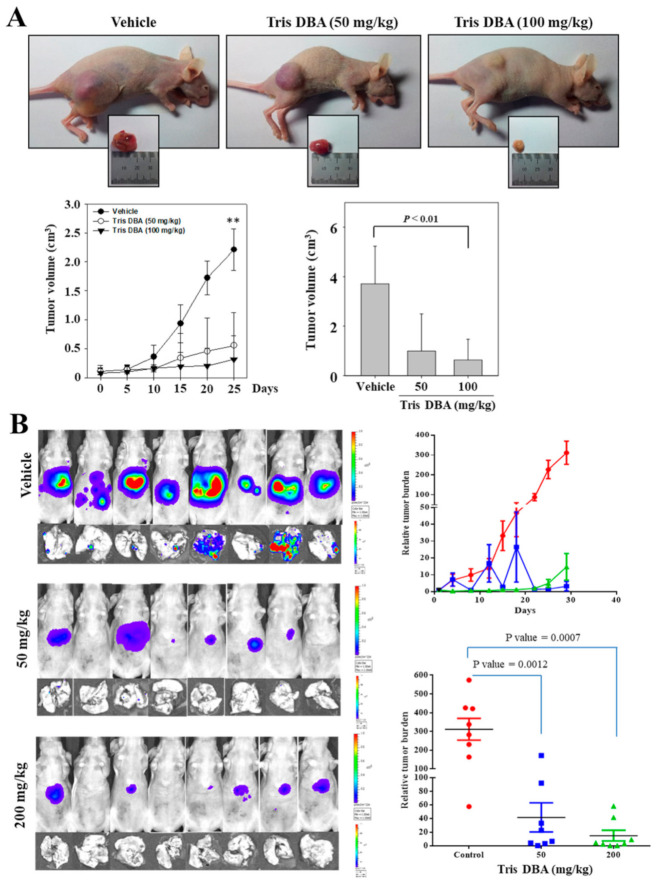
Tris DBA induces antitumor activity in xenograft MM and the orthotopic HCC mice model. (**A**) U266 cells were subcutaneously injected into xenograft tumors. After tumors reached 0.25 cm in diameter, the mice were divided into three groups (n = 6/group) and administered with 50 mg or 100 mg/kg body weight. The tumor volume was monitored throughout the study tenure. (**B**) HCCLM3-Luc cells-induced tumors are orthotopically implanted to the liver tissue followed by treatment with 0.1% DMSO (*n* = 7) or Tris DBA (*n* = 7) (administered 50 mg/kg or 200 mg/kg intraperitoneally, thrice a week, for four weeks). The tumor progression/regression was monitored twice a week by quantifying the bioluminescence intensity and the graph is plotted using these values. The scattered plot indicates the tumor burden that was quantified by measuring photon counts before the first administration of Tris DBA and at the last dose. Unpaired t-test with Welch’s correction. Bars indicate standard deviation; ** *p* < 0.01.

We investigated the anticancer activity of Tris DBA in xenograft MM and the orthotopic HCC mice models. We subcutaneously implanted U266 cells to the right flank of six weeks old athymic *nu/nu* female mice to establish the xenograft tumor model. When tumor size attained 0.25 cm, the mice were randomly divided into three groups (*n* = 6). The first group served as a control. The second and third groups received an intraperitoneal injection of Tris DBA (50 mg/kg thrice a week and 100 mg/kg thrice a week, respectively). At the end of four weeks, we observed a significant reduction in the volume of tumors in the 100 mg/kg treated group (*p* < 0.01) (Figure 7A).

To verify the antitumor effect of Tris DBA on solid tumors, we next established an orthotopic HCC mouse model as described earlier and randomly distributed it into three groups. The first group was used as a control. The second and third group animals were intraperitoneally injected with Tris DBA (50 mg/kg thrice a week and 200 mg/kg thrice a week, respectively) for four weeks and the tumor burden was measured by the non-invasive bioluminescence technique. Tris DBA significantly inhibited tumor growth in both groups (*p* = 0.0007) (Figure 7B).

### 3.13. Tris DBA Reduced the Number of Ki67+ Cells in Tumor Tissues from the Xenograft MM Mouse Model

**Figure 8:**
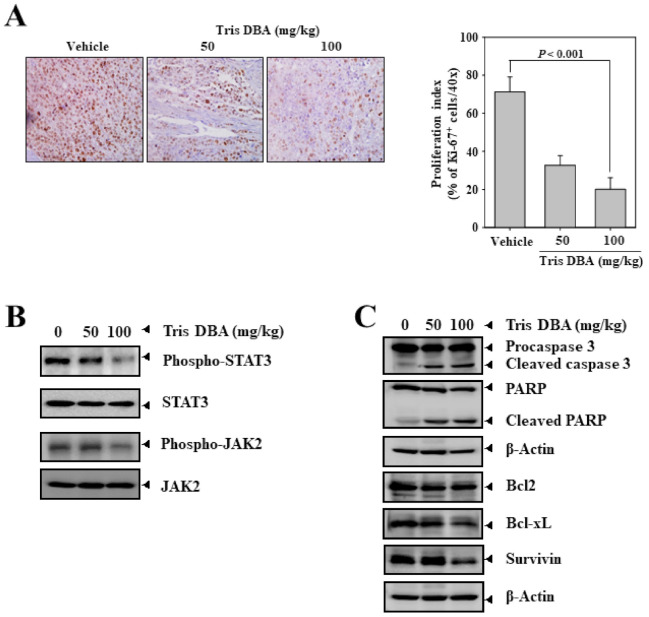
Tris DBA decreases the activation of STAT3 and its regulatory proteins as well as the levels of its downstream proteins. (**A**) MM tumor tissue from Tris-DBA treated and the untreated group was used for examination of the expression of Ki-67 by immunohistochemistry analysis. (**B**,**C**) The expression of STAT3 signaling pathway proteins and apoptotic markers was examined in MM tumor tissues.

We next processed the MM tumor tissue samples and performed immunohistochemical analysis to determine the proliferation index. Tumor tissues from Tris DBA treated mice displayed a marked reduction in the levels of Ki-67^+^ cells in a dose-dependent fashion (Figure 8A). These results suggest that Tris DBA may also induce its antitumor activities by reducing the proliferation of cancer cells.

### 3.14. Tris DBA Repressed the Activation of STAT3 Signaling Proteins in Tumor Tissues

We further analyzed the phosphorylation status of STAT3, JAK1, and JAK2 proteins in the tumor tissues of the xenograft MM mouse model. We observed a substantial reduction in the phosphorylation of STAT3, JAK1, and JAK2 in the tumor tissues of mice treated with 100 mg/kg of Tris DBA (Figure 8B). These results establish that the lowering of tumor burden by Tris DBA could be due to the abrogation of the STAT3 signaling pathway.

### 3.15. Tris DBA Modulated the Expression of Apoptosis-Related Proteins

We profiled the apoptosis-related proteins (Bcl-2, Bcl-xL, Survivin, PARP, and procaspase 3) in tumor tissues of the xenograft MM mouse model. We observed a marked decrease in Bcl-2, Bcl-xL, Survivin, full-length PARP, and procaspase-3 (Figure 8C), thus indicating that Tris DBA can interfere with tumor development by inducing apoptosis in tumor cells.

## 4. Discussion

Aberrant activation of STAT3 is often observed in numerous human malignancies, including MM, leukemias, lymphomas, breast, ovary, melanoma, glioma, head and neck, liver, and kidney cancers [54,55,56]. A few studies have demonstrated that deregulated STAT3 activation has not been observed in the normal tissues adjacent to tumors [57,58]. Additionally, the expression of STAT3 in tumor tissue serves as a predictive marker of prognosis, and elevated STAT3 expression is associated with a reduced prognostic ability and worse three-year overall survival rate of human solid tumors [59,60,61,62]. The constitutive activation of STAT3 can be controlled by various mechanisms, including the loss of negative regulators (PTPs, SOCS, and PIAS), the formation of positive feedback mechanisms in the tumor microenvironment, elevated activity of upstream kinases, and somatic mutations in the STAT3 gene [63]. For instance, elevated levels of IL-6 are produced due to a positive feedback mechanism in the tumor microenvironment, which may regulate the persistent phosphorylation of STAT3 signaling in human cancers [64,65,66]. We have previously demonstrated the anticancer potential of several natural and synthetic compounds in various disease models [67,68,69,70,71]. Herein, we have evaluated the action of Tris DBA on the STAT3 signaling in HCC and MM cell-based studies and preclinical settings. Tris DBA was initially described as an inhibitor of N-myristoyltransferase-1 and here we have comprehensively demonstrated its mode-of-action in two different tumor models. Tris DBA decreased the viability of both types of cancer cell lines (HCC and MM) and caused a pronounced inhibition of STAT3 activation. DNA undergoes fragmentation in the cells that are committed to apoptosis due to activation of caspases and thus they are known as hypodiploid cells, which are marked as subG1 cell population. We observed an increased amount of the subG1 cell population on treatment with Tris DBA in the flowcytometric analysis. The pro-apoptotic actions of the drug were further confirmed by FITC conjugated Annexin-V-PI staining. To understand the underlying molecular mechanism of Tris DBA-induced cytotoxicity, we evaluated the potential of Tris DBA to modify STAT3 phosphorylation in tumor cells and noticed a marked decline in the activation of STAT3 in all the tested cell lines. 

An enormous number of studies have reported that STAT3 phosphorylation is essential for its translocation into the nucleus to express the target genes [72,73,74,75]. In contrast to our findings, Kay and colleagues have demonstrated that Tris DBA did not significantly inhibit the activation of STAT3 in primary chronic lymphocytic leukemia B-cells [38]. However, we hypothesized that the cytotoxic efficacy of Tris DBA could be due to the alteration of the STAT3 signaling cascade. We next evaluated the effect of Tris DBA on IL-6 stimulated STAT3 phosphorylation. RPMI-8226 cells lack persistently activated forms of STAT3 and IL-6 can function to promote the activation of STAT3 signaling [47,76]. The bone marrow microenvironment contains several growth factors along with IL-6 released by the bone matrix and osteoblasts, creating an ideal situation for the promotion of oncogenesis in various cancers including MM [77]. Therefore, it is apt to have an inhibitor that can counteract the effect of IL-6 induced signaling. We observed a significant decrease in IL-6 driven STAT3 phosphorylation in MM cells. 

Phosphorylation of STAT3^Y705^ is crucial for its translocation to the nucleus to transcribe the downstream genes. The substitution of tyrosine with phenylalanine using site-directed mutagenesis can cause a substantial decrease of PIAS3-STAT3 complex and their nuclear localization despite EGF-induction [78]. Tris DBA downregulated the levels of nuclear STAT3 and suppressed the DNA binding ability of nuclear STAT3. This could be due to the inhibition of STAT3 phosphorylation, which is essential for nuclear localization and target gene expression. We next determined the action of Tris DBA on the activation status of STAT3 upstream NRTKs such as JAK1, JAK2, and Src in MM and HCC cells. Phosphorylation of these kinases was inhibited in both types of cancer cells. Moreover, cytokine-induced activation of these NRTKs was also downregulated, indicating that the abrogation of STAT3 phosphorylation may be caused by the inhibition of upstream signaling partner proteins. 

The phosphorylation state of STAT3 signaling proteins can also be regulated by a class of protein tyrosine phosphatases (PTPs), including SHP1, SHP2, PTPεC, PTP1B, and CD45 [79,80,81]. Therefore, we treated U266 cells with Tris DBA and pervanadate, a broad range phosphatase inhibitor, and tested for the activation of STAT3. Interestingly, pervanadate treatment reversed the Tris DBA-driven STAT3 inhibition. This indicated that PTP is involved in the Tris DBA-induced STAT3 signaling inhibition. We treated U266 cells again with pervanadate and Tris DBA and analyzed the expression of major PTPs, such as SHP1, SHP2, and PTP1B. We observed a substantial enhancement in the levels of both SHP2 mRNA and protein. The treatment of U266 cells with SHP2-directed siRNA resulted in the loss of SHP2 protein and further treatment of these cells with Tris DBA displayed no effect on STAT3 phosphorylation. However, further investigation may be required to understand the relationship between the expression of SHP2 and Tris DBA in MM cells. Not surprisingly, SHP2 suppressed the proliferation of esophageal squamous cell cancer by inhibiting STAT3 phosphorylation, and depletion of SHP2 resulted in the attenuation of cisplatin sensitivity [82]. Moreover, Chong and colleagues reported that IL-6 mediated STAT3 activation led to the transcriptional activation of PRL-3 (an oncogenic phosphatase overexpressed in MM) and PRL-3 increased the rephosphorylation of STAT3 via the direct interaction and deactivation of SHP2 forming a feedforward loop in MM [83]. 

Bortezomib is the first proteasome inhibitor approved by the US FDA for the treatment of various malignancies [84]. Li and colleagues reported that bortezomib can increase the expression of phosphorylated STAT3 and STAT3 in head and neck squamous cell carcinoma cells, which may contribute to a substantial reduction in its therapeutic efficacy, and co-treatment of bortezomib with a STAT3 inhibitor has been suggested for better anticancer effects [85]. Guan and coworkers have further demonstrated that bortezomib resistance in MM may be partly mediated through activated STAT3 signaling [86]. We tested the potency of Tris DBA towards bortezomib-resistant RPMI-8226 cells. Tris DBA showed relatively less cytotoxicity compared to bortezomib-resistant RPMI-8226 cells compared with bortezomib-sensitive RPMI-8226 cells. Tris DBA also substantially decreased the levels of oncogenic proteins, including Mcl-1, cyclin D1, and Bcl-2, in all the tested cell lines. During apoptosis, procaspase 3 undergoes cleavage on receiving signals from activated caspase 8 and 9 to induce apoptosis [87]. We observed a marked cleavage of procaspase-3 and downstream effector PARP, indicating that cells are undergoing apoptosis on treatment with Tris DBA.

STAT3 can also regulate the activation of key transcription factors such as Snail and Twist which mediate the epithelial-mesenchymal transition (EMT) process, and in turn, metastasis of cancer cells to the distant organs [88]. IL-6 has been reported to activate STAT3 to promote EMT through the induction of Snail expression in cancers [89,90]. Phosphorylated STAT3 also triggered the expression of the *Twist* gene to promote oncogenic functions [91]. Next, we determined the efficacy of Tris DBA on the invasion and migration of cancer cells. Tris DBA significantly blocked the cell motility in both the assay systems. The inhibition of migration and invasion could be due to the suppression of the STAT3-dependent EMT process.

Interestingly, Tris DBA was observed to be non-toxic (up to 200 mg/kg body weight) in acute toxicity studies conducted in mice. Next, we examined its antitumor potential in xenograft MM and orthotopic HCC mouse models. Tris DBA displayed significant antitumor activities and it effectively abrogated the tumor progression in both the preclinical models. It has been also reported that Tris DBA did not induce local nor systemic toxicity at 40 mg/kg/day in the A375 melanoma mouse model [36]. Importantly, Tris DBA downmodulated the expression of activated JAK1, JAK2, and STAT3, and Ki-67 in MM tumor tissues. These results are consistent with the outcome of our various cell-based assays.

Although Tris DBA has been demonstrated to inhibit STAT3 signaling and induce cytotoxicity in cancer cell lines in an in vivo model, previous studies have shown that Tris DBA can also regulate the activity of many cancer-related proteins (such as MAPK, Akt, STAT3, phospho-S6 kinase, and N-myristoyltransferase-1). Moreover, Tris DBA also modulated the mRNA and protein expression of SHP2. Previous reports also suggest that PRL-3 (a STAT3 driven phosphatase coding gene) directly interacts with SHP2 to counteract its activity. The underlying mechanism behind the transcriptional control of SHP2 remains a question yet to be answered. These studies indicate that there is a much more complex mechanism involved in the Tris DBA mediated cytotoxicity than evinced by the present-day understanding. We are of the opinion that Tris DBA does not impart antioncogenic effects solely through the abolishment of the STAT3 pathway. Certainly, Tris DBA mediates its anticancer effects at least partly via repression of the STAT3 pathway.

## 5. Conclusions

Tris DBA has been demonstrated as a potent inhibitor of the STAT3 signaling cascade in MM and HCC cell lines and preclinical models for the first time. It can be reconciled that Tris DBA is a broad-spectrum anticancer agent and further research may be warranted to completely understand its off-targets and its possible potential clinical application.

## Data Availability

All the data associated with this study is freely available.

## Supplementary Materials

The following are available online at https://www.mdpi.com/article/10.3390/cancers13215479/s1, Figure S1: the uncropped western blot figures.
